# Intrinsic hypoxia sensitivity of the cytomegalovirus promoter

**DOI:** 10.1038/cddis.2015.259

**Published:** 2015-10-15

**Authors:** K Wendland, M Thielke, A Meisel, P Mergenthaler

**Affiliations:** 1 Department of Experimental Neurology, Center for Stroke Research Berlin, Charité Universitätsmedizin Berlin, Berlin, Germany; 2 NeuroCure Cluster of Excellence, Charité Universitätsmedizin Berlin, Berlin, Germany; 3 Department of Neurology, Center for Stroke Research Berlin, Charité Universitätsmedizin Berlin, Berlin, Germany; 4 Biological Sciences, Sunnybrook Research Institute, University of Toronto, Toronto, ON, Canada

*Dear Editor,*

Here, we report that the cytomegalovirus (CMV) promoter exhibits intrinsic hypoxia sensitivity. Complex and refined genetic cellular or animal models of disease enabling targeted expression of transgenes in cellular subtypes such as select groups of neurons in the brain are now routinely developed and allow investigation of distinct mechanisms in cell death and other fundamental cellular pathways. Thus, near-physiological expression of transgenes is imperative and deserves close attention in the generation or application of these models. However, systematic information of how stress conditions affect promoter function and thus protein expression is scarce.

Here, we use different fluorescent proteins with different turnover kinetics to illustrate that the sensitivity of a promoter to the conditions of the cellular environment, such as oxygen levels, can mask or mimic dynamics or function of transgene expression. We found that over the course of cultivation there was a marked decrease in fluorescence intensity of the green fluorescent protein eGFP (enhanced green fluorescent protein)- or destabilized (d2)eGFP-transfected primary rat brain cortical neurons after 4–7 days in culture. After 10 days most transfected neurons showed nearly undetectable green fluorescence when d2eGFP (Figure 1a–i) or eGFP (data not shown) were expressed under the control of the CMV promoter. This time course corresponds well with the stability of eGFP in cells of about 1 week.^1^ Furthermore, 24 h after hypoxia (see Mergenthaler *et al.*,^2^ for details), there was a marked increase in green fluorescence in transfected neurons (Figure 1a–i and data not shown). Likewise, protein levels for d2eGFP were nearly undetectable by western blotting before hypoxia, but increased to readily detectable levels thereafter (Figure 1b–i). In contrast, d2eGFP was expressed at constant high levels when driven by the human ubiquitin promoter (UBI), without apparent increase of green fluorescence after hypoxia (Figure 1a and b). As a sign of cellular damage after hypoxia, protein levels under these conditions rather slightly decreased (Figure 1a and b). Moreover, other ubiquitous and neuron-specific promoters (CAG, composite chicken *β*-actin promoter with CMV enhancer; FER, human ferritin promoter; C1.3, long version of the calcium/calmodulin-dependent protein kinase II promoter) allowed constant expression of the monomeric red-fluorescent protein tagRFP targeted to mitochondria over the entire culture period, whereas CMV-driven expression resulted in nearly undetectable protein levels by western blotting and increased expression after hypoxia (Figure 1c). In contrast, the tetrameric red-fluorescent protein DsRed2 under the control of the CMV promoter showed stable fluorescence intensity over the normal neuronal culture period of 10 days (data not shown), which corresponds to the greater stability of this red-fluorescent protein in cells.^1^ These data indicate that hypoxia sensitivity is not dependent on the species from which the fluorescent protein was derived. Of note, we also found reactivation of CMV-driven expression of d2eGFP after 90 min oxygen–glucose deprivation (OGD) or oxygen deprivation at 0.3% O2, but not after glucose deprivation (GD, 90 and 180 min, data not shown). In summary, these data indicate that hypoxia is a central stimulus for reactivation of CMV-driven transgene expression.

Silencing of the CMV promoter in the central nervous system, in neurons, and in other cells has been well characterized.^3, 4^ In addition, efforts have been made to introduce hypoxia sensitivity to the CMV promoter by coupling it to hypoxia response elements (HREs).^5^ However, in this report we demonstrate that the CMV promoter is intrinsically hypoxia sensitive and can be reactivated after a brief period of hypoxia without the need for additional HREs. Likewise, reactivation of silenced CMV promoter has been shown for cellular stress induced by lipopolysaccharide or physical stress^6^ and pharmacological agents such as histone deacetylase inhibitors.^7^

Transgene expression under the control of the CMV promoter continues to play a big role in cell death research and other fields of cell biology for the investigation of fundamental cellular functions. Our data emphasize that care should be taken when interpreting results using the CMV promoter in different models of cellular stress and in particular when proteins are expressed without a tag to distinguish exogenous and endogenous protein turnover. Finally, the CMV promoter might not be suited to function as a constitutive promoter for constant expression of transgenes.

## Figures and Tables

**Figure 1:**
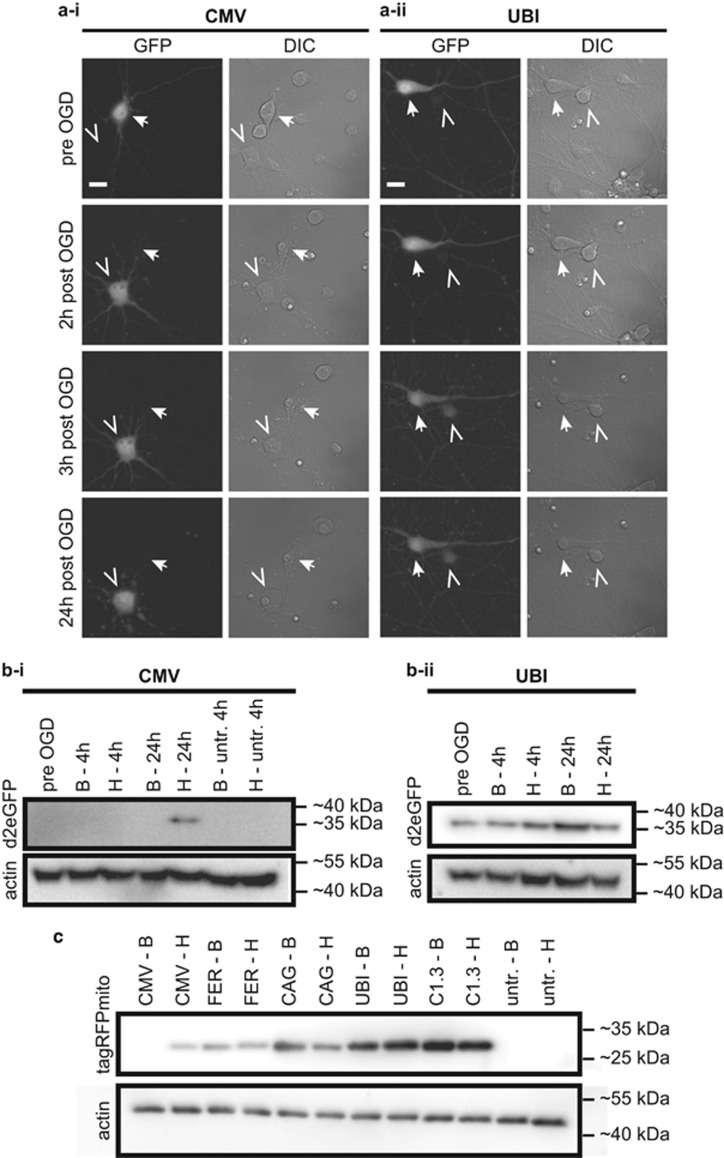
Intrinsic hypoxia sensitivity of the CMV promoter. (**a**) Primary embryonic rat brain cortical neurons were transfected with a plasmid encoding for destabilized (d2)eGFP under the control of the CMV (a-i) or UBI (a-ii) promoter and imaged before and at several time points after OGD. The closed arrow in a-i marks a cell that shows weak green fluorescence before OGD and loses its fluorescence after cell death during the experiment, the open arrowhead marks a cell that is non-fluorescent before OGD but shows bright green fluorescence rapidly after OGD. The closed arrow in a-ii shows a cell with bright green fluorescence before OGD which dies in the course of 24 h after OGD and thereby decreases in fluorescence intensity. The open arrowhead in a-ii marks a cell that has weak green fluorescence throughout the experiment. Note that the apparent increase in fluorescence is due to the decrease of fluorescence intensity of the neighboring cell. DIC, differential interference contrast. Scale bar=15 *μ*m. (**b**) D2eGFP levels were undetectable by western blotting until 24 h after OGD when expressed under the control of the CMV promoter (b-i), whereas d2eGFP was readily detectable at all time points when expressed under the control of the UBI promoter (b-ii). B, baseline (control conditions); H, hypoxia/OGD (**c**) Mitochondrially targeted tagRFPMito was almost undetectable until 24 h after OGD when expressed from the CMV promoter. Expression from FER, composite CAG, UBI or calcium/calmodulin-dependent protein kinase II (C1.3) promoters did not result in significantly altered expression of the mitochondrial red-fluorescent protein. Note that only very high overexposure or adjustment of the contrast of this blot shows a faint band for CMV-tagRFPMito before OGD (not shown here), and that the band intensity for UBI-tagRFPMito after hypoxia (H) is slightly higher than before (possibly due to its subcellular localization or variable protein expression). B, baseline (before hypoxia); H, 24 h after hypoxia (OGD). In all experiments neuronal cultures, transfections and OGD experiments were essentially performed as described.^2^ OGD or oxygen deprivation (OD) experiments were performed in a hypoxia workstation (Ruskinn Concept400 or InVivo2) with Bss_0_ (116 mM NaCl, 5.4 mM KCl, 0.8 mM MgSO_4_, 1 mM NaH_2_PO_4_, 26.2 mM NaHCO_3_, 10 *μ*M glycine, 1.8 mM CaCl_2_, 10 mM Hepes; 37 °C, 0% or 0.3% O_2_, 5% CO_2_) for OGD and Bss_10_ (Bss_0_+10 mM glucose) for OD (37 °C, 0.3% O_2_, 5% CO_2_). For GD (37 °C, 21% O_2_, 5% CO_2_) Bss0 was used. Microscopy of d2EGFP constructs in 8 well *μ*-slides (Ibidi, Martinsried, Germany) was performed at the same position in each well using a mark and find software (Leica LAS AF, Wetzlar, Germany) for all time points. Thereby, it was possible to image changes in d2EGFP expression pattern in the exact same cell before and after OGD, OD or GD. Variations in image positions are due to *μ*m-misplacement of the 8 well *μ*-slides in the microscopy stage. For figure preparation, 16 bit TIF images were converted to 8 bit TIF and contrast was adjusted uniformly after background subtraction with a rolling ball algorithm in ImageJ (Fiji v. 1.47d, http://www.fiji.sc). Plasmids were generated by subcloning d2eGFP (Clontech, Takara Bio Europe, St-Germain-en-Laye, France) or TagRFP-mito (Evrogen, Moscow, Russia) into vector backbones containing the CMV (pCDNA3.1, Invitrogen), UBI (Addgene plasmid 11651), CamKII1.3 (C1.3, Addgene plasmid 32577), or FER (pVitro2-neo-mcs, Invivogen) promoters
